# Temporal pattern of tuberculosis cure, mortality, and treatment abandonment in Brazilian capitals

**DOI:** 10.1590/1518-8345.3019.3218

**Published:** 2019-12-05

**Authors:** George Jó Bezerra Sousa, Thiago Santos Garces, Maria Lúcia Duarte Pereira, Thereza Maria Magalhães Moreira, Germana Maria da Silveira

**Affiliations:** 1 Universidade Estadual do Ceará, Departamento de Enfermagem, Fortaleza, CE, Brazil.; 2 Bolsista da Fundação Cearense de Apoio ao Desenvolvimento Científico e Tecnológico, Fortaleza, CE, Brazil.; 3 Bolsista da Coordenação de Aperfeiçoamento de Pessoal de Nível Superior (CAPES), Brazil.

**Keywords:** Tuberculosis, Epidemiology, Time Series Studies, Treatment Adherence and Compliance, Treatment Outcome, Nursing, Tuberculose, Epidemiologia, Estudos de Séries Temporais, Cooperação e Adesão ao Tratamento, Resultado do Tratamento, Enfermagem, Tuberculosis, Epidemiología, Estudios de Series Temporales, Cumplimento y Adherencia al Tratamento, Resultado del Tratamento, Enfermería

## Abstract

**Objective:**

to analyze the temporal pattern of tuberculosis cure, mortality, treatment abandonment in Brazilian capitals.

**Method:**

this is an ecological study whose data source was the Information System of Notifiable Diseases for Tuberculosis (*Sistema de Informação de Agravos de Notificação para Tuberculose*). For analysis of temporal evolution, regressions by join points were performed considering the annual percentage variation and the significance of the trend change with 95% confidence interval.

**Results:**

542,656 cases of tuberculosis were found, with emphasis on a 3% decrease per year in the cure rate for Campo Grande (interval: −5.0 - −0.9) and a 3.5% increase for Rio de Janeiro (interval: 1.9 - 4.7). Regarding abandonment, it decreased 10.9% per year in Rio Branco (interval: −15.8 - −5.7) and increased 12.8% per year in Fortaleza (interval: 7.6 - 18.3). For mortality, a decreasing or stationary tendency was identified, with a greater decrease (7.8%) for Porto Velho (interval:−11.0 - −5.0) and a lower one (2.5%) in Porto Alegre (interval:−4.5 - −0.6).

**Conclusion:**

the rates of cure and abandonment are far from the ones recommended by the World Health Organization, showing that Brazilian capitals need interventions aimed at changing this pattern.

## Abstract (pt)

**Objetivo:**

analisar o padrão temporal dos desfechos cura, abandono e mortalidade da tuberculose em capitais brasileiras.

**Método:**

estudo ecológico cuja fonte de dados foi o Sistema de Informação de Agravos de Notificação para tuberculose. Para análise da evolução temporal foram realizadas regressões por joinpoints considerando a variação percentual anual e significância da mudança de tendência com 95% de confiança.

**Resultados:**

registraram-se 542.656 casos de tuberculose, com destaque de 3% de decréscimo ao ano na taxa de cura para Campo Grande (intervalo: -5,0 - -0,9) e acréscimo de 3,5% para o Rio de Janeiro (intervalo: 1,9 - 4,7). Em relação ao abandono, enfatiza-se redução de 10,9% ao ano em Rio Branco (intervalo: -15,8 - -5,7) e aumento de 12,8% ao ano em Fortaleza (intervalo: 7,6 - 18,3). Para a mortalidade, identificou-se tendência decrescente ou estacionária, evidenciando maior diminuição para Porto Velho 7,8% (intervalo: -11,0 - -5,0) e menor em Porto Alegre 2,5% (intervalo: -4,5 - -0,6).

**Conclusão:**

as taxas de cura e abandono encontram-se distantes do preconizado pela Organização Mundial da Saúde, evidenciando capitais que necessitam de intervenções direcionadas para mudança desse padrão.

## Abstract (es)

**Objetivo:**

analizar el patrón temporal de los desenlaces curación, abandono y mortalidad de la tuberculosis en capitales brasileñas.

**Método:**

estudio ecológico cuya fuente de datos fue el Sistema de Información en Salud para tuberculosis. Para el análisis de la evolución temporal, se realizaron regresiones por joinpoints teniendo en cuenta la variación porcentual anual y la significancia del cambio de tendencia con el 95 % de confianza.

**Resultados:**

se registraron 542.656 casos de tuberculosis, y se resaltan una reducción del 3 % al año en la tasa de curación en Campo Grande (intervalo: -5,0 - -0,9) y un aumento del 3,5 % en Río de Janeiro (intervalo: 1,9 - 4,7). Respecto al abandono, se resaltan una reducción del 10,9 % al año en Rio Branco (intervalo: -15,8 - -5,7) y un aumento del 12,8 % al año en Fortaleza (intervalo: 7,6 - 18,3). Para la mortalidad, se identificó una tendencia decreciente o estacionaria, lo que pone de manifiesto una reducción mayor, de un 7,8 %, en Porto Velho (intervalo: -11,0 - -5,0), y menor, de un 2,5 %, en Porto Alegre (intervalo: -4,5 - -0,6).

**Conclusión:**

las tasas de curación y abandono se encuentran distantes de lo que aboga la Organización Mundial de la Salud, lo que hace patente la necesidad de realizar intervenciones direccionadas en algunas capitales para cambiar este patrón.

## Introduction

Worldwide, tuberculosis (TB) is the ninth cause of death and the first one by infectious disease. In 2017, 10 million new cases and 1.3 million deaths caused by this infection were estimated, showing a reality that is inconsistent with what is foreseen in the Sustainable Development Goals (SDG), which guide the reduction of the TB epidemic^(^ 1 ^-^ 2 ^)^.

Specifically for TB, the SDG recommend prioritizing the incidence reduction by 80% and its deaths by 90% up to 2030. In addition to the SDGs aimed at reducing the outcomes related to TB, we have the End TB Strategy, created by the World Health Organization (WHO), which expands this deadline to 2035, expanding the incidence reduction to 90% and deaths to 95%^(^ 2 ^-^ 5 ^)^. The WHO also recommends a minimum 85% cure and a maximum 5% treatment abandonment^(^ 6 ^-^ 7 ^)^.

The End TB Strategy is based on the principles of “integrated care and prevention, focused on the patient; bold policies and support systems, with emphasis on the social protection of vulnerable populations; and intensification of research and innovation.” However, obstacles that impede the achievement of the proposed goals regarding TB still have to be overcome, especially in emerging countries. In this perspective, in 2017, Brazil signed a commitment term to develop a research agenda in the area of TB^(^ 4 ^-^ 5 ^)^.

Brazil is on the list of countries that amount to 49% of the world TB burden and 60% of its resistant forms, and, even with an annual decrease of 1.7% in the TB incidence coefficient, it currently has a national average of 32.4/100,000 inhabitants^(^ 8 ^)^, which makes the constant verification of TB data urgent. Thus, this study presents a large temporal series was performed for the outcomes (cure, abandonment, and mortality) by TB in Brazilian capitals, by their population aggregation and data completeness in the bases, which makes the evidence generated have greater external validity. In addition, clusters are determinant in the TB transmission chain^(^ 9 ^)^.

One can find in the literature studies on TB temporal patterns. However, they address only one capital (or specific region) and mainly investigate incidence or prevalence^(^ 10 ^-^ 13 ^)^. This investigation includes three important outcomes (cure, abandonment, and mortality), which are seen in a large temporality, population, and statistical sensitivity.

Studies with these characteristics have been pointed out as important^(^ 10 ^-^ 13 ^)^, as they may show results that fill gaps, such as those related to Brazil achieving the goals set to reduce negative outcomes by TB. In this study, one can have a broad view on how cure and abandonment may affect mortality.

The literature endorses the need to fill knowledge gaps about tuberculosis treatment outcomes as a strategy for nursing care and health management, mainly focused on people with a chance to present negative results^(^ 10 ^)^. Thus, this study aimed to analyze the temporal pattern of the outcomes cure, abandonment, and mortality by TB in Brazilian capitals.

## Method

This is an ecological study of time series, which analyzed three outcomes of TB treatment (cure, abandonment, and death) in all Brazilian capitals, from 2001 to 2015. They were selected because they are important indicators of disease management and have greater completeness in the Information System of Notifiable Diseases for Tuberculosis (Sinan-TB). Drug resistance was not included because of the expressive number of missing data over several years; transfer was not included because it was not within the scope of this investigation; and other outcomes, such as failure and primary abandonment, were only included in the follow-up form from 2015 on, making it impossible to analyze them in this study.

This study was conducted from March to June 2018. Three information systems from the Brazilian Ministry of Health were used: Sinan-TB, the Mortality Information System (SIM), and population projections of each year, according to the Brazilian Institute of Geography and Statistics (IBGE). It should be emphasized that the terms cure and abandonment are in accordance with the follow-up and termination form of TB treatment, as well as mortality was used during the study for calculating such coefficient.

The data for the systems previously exposed were obtained in the website of the Informatics Department of SUS (Datasus) by the online application TabNet. The total number of cases in each year was searched, and then cure or abandonment were selected as outcome situation to include them in the calculation of the rate of both indicators (Sinan-TB). After that, data on vital statistics were searched by SIM. We selected the option of general mortality per group of the 10th International Classification of Diseases (ICD-10), where we were able to select TB as both the underlying and associated cause. Finally, the population data were accessed in the demographic and sociodemographic information tab of IBGE, and it was possible to choose the population living in the selected city in each year of the time series.

Despite conducting the time series up to 2015 for mortality, the authors opted to stop the series in 2014 for the outcomes cure and abandonment because of the high incompleteness in the database from that date on. The data were made available online and the files were downloaded in CSV format. The raw data of the disease in each year and its outcomes were tabulated in Excel spreadsheet and imported into the free software Joinpoint Regression Program version 4.6.0.0^(^ 14 ^)^. This software was created to analyze the tendency of cancer, but is currently used in other fields of epidemiology, due to its statistical potentiality in analyzing temporal patterns^(^ 11 ^,^ 15 ^-^ 16 ^)^.

The analysis that the program performs is segmented linear, with logarithmic transformation of the values. A researcher tests if one or more points should be added to the linear model by the Monte Carlo permutation. That is, one evaluates whether a multiple segment line describes the model better than only a straight line. For this reason, the analysis is also known as regression by inflection points. The Annual Percentage Change (APC) was calculated, with a 95% confidence interval (95%CI), in which a negative value of APC indicates a decreasing trend and a positive value, an increasing trend^(^ 11 ^,^ 15 ^-^ 16 ^)^. At the end of the period, it was possible to obtain the Average Annual Percentage Change (AAPC), which shows how the change occurred during the period studied. If there is more than one inflection point, the AAPC will consider all of them for its calculation, otherwise the AAPC will be equal to the APC.

Each inflection point added to the model means a change of the linear trend, that is, it could be expressed only by a straight line or its change would indicate the insertion of an inflection point with the inclusion of a new line segment to the time series. The model was adjusted assuming that the number of inflection points could vary from zero (only one segment) to two (three segments) over the years. A 5% significance level was established to test the null hypothesis that the APC and the AAPC of the series are equal to zero^(^ 17 ^)^. Thus, both for the analysis of APC and AAPC, the results with p<0.05 or only positive (increasing trend) or only negative (decreasing trend) 95%CI are significant.

The year of occurrence was defined as independent variable and the percentage of people with the disease in each year was defined as dependent variable, both calculated directly in the program and standardized according to the previously exposed criteria of logarithms. For this, the number of cases was included with the selected outcome as numerator and the total number of cases as denominator. However, for the outcome mortality, the number of deaths by TB was selected as numerator and the population of the year was used as denominator. The values of cure and abandonment were analyzed in percentage and, for mortality values, the coefficient was considered for 100,000 inhabitants. The presence of lacking data was an exclusion factor in these analyses.

According to Resolution 510 of 2016 of the National Health Council, public domain data do not need to be assessed by the Ethics Committee System.

## Results

Among the incident and prevalent cases in the period from 2001 to 2014, 542,656 cases of TB were reported in the 27 cities. In general, the capitals presented a 60.1% (n=326.478) cure rate and a 13.6% (n=73.867) abandonment rate.

In the period of the series, Boa Vista had the highest average cure rate (78.8%) and Teresina had the lowest one (48.3%); interestingly, Teresina was the capital with the lowest rate of treatment abandonment (4.1%), and Porto Alegre had the highest one (19.5%). Regarding mortality, Recife presented the highest average coefficient (8.2/100,000 inhabitants) of the period and Brasília, the lowest one (0.6/100,000 inhabitants). The data referring to the average rate for the three outcomes in each year can be seen in Table 1.

**Table 1:** Average rates of cure, abandonment, and mortality for the treatment of TB* in Brazilian capitals from 2001 to 2015

Capitals	Cure (95%CI)^†‡^	Abandonment (95%CI)^†‡^	Mortality (95%CI)^†§^
North Region			
Porto Velho	65.2 (61.8; 68.5)	14.9 (12.7; 17.1)	2.8 (1.9; 3.7)
Rio Branco	79.4 (74.4; 84.3)	8.0 (5.3; 10.7)	3.6 (2.6; 4.6)
Manaus	70.5 (67.6; 73.5)	12.2 (11.2; 13.2)	4.0 (3.7; 4.4)
Boa Vista	78.8 (75.6; 81.9)	5.0 (3.9; 6.1)	1.3 (0.7; 1.8)
Belém	66.9 (64.7; 69.1)	12.6 (11.6; 13.6)	5.1 (4.5; 5.8)
Macapá	68.7 (64.4; 73.0)	12.2 (10.5; 13.8)	1.3 (1.0; 1.6)
Palmas	71.3 (65.2; 77.4)	5.3 (2.3; 8.3)	0.8 (0.5; 1.1)
Northeast Region			
São Luís	67.8 (65.8; 69.7)	14.3 (12.2; 16.5)	4.2 (3.8; 4.6)
Teresina	48.3 (41.0; 55.5)	4.1 (3.4; 4.8)	2.2 (1.8; 2.5)
Fortaleza	60.5 (54.1; 67.0)	14.4 (11.3; 17.5)	4.2 (3.7; 4.6)
Natal	57.8 (54.2; 61.4)	13.7 (11.6; 15.9)	2.6 (2.3; 3.0)
João Pessoa	60.3 (57.2; 63.5)	14.8 (12.4; 17.2)	2.4 (2.1; 2.7)
Recife	52.3 (50.9; 53.8)	15.4 (14.1; 16.6)	8.2 (7.4; 9.0)
Maceió	63.4 (60.7; 66.2)	15.1 (14.2; 16.0)	4.6 (4.2; 5.0)
Aracaju	65.9 (62.1; 69.6)	15.6 (13.5; 17.8)	2.1 (1.8; 2.5)
Salvador	58.1 (55.6; 60.6)	8.9 (8.1; 9.6)	4.4 (3.8; 5.0)
Southeast Region			
Belo Horizonte	61.4 (59.9; 63.0)	17.9 (16.3; 19.6)	1.4 (1.2; 1.6)
Vitória	66.5 (62.2; 70.8)	8.3 (7.1; 9.5)	2.5 (1.9; 3.1)
Rio de Janeiro	51.7 (46.1; 57.3)	12.7 (10.8; 14.5)	6.2 (5.9; 6.5)
São Paulo	68.4 (66.0; 70.8)	15.8 (15.3; 16.4)	3.2 (2.9; 3.5)
South Region			
Curitiba	64.2 (60.1; 68.3)	10.9 (9.6; 12.2)	1.2 (1.0; 1.5)
Florianópolis	54.0 (48.4; 59.5)	13.8 (12.4; 15.2)	1.1 (0.8; 1.3)
Porto Alegre	51.8 (50.1; 53.6)	19.5 (15.8; 23.1)	4.4 (4.0; 4.9)
Midwest Region			
Campo Grande	60.9 (55.1; 66.7)	8.8 (6.9; 10.7)	1.7 (1.4; 2.0)
Cuiabá	70.4 (66.8; 74.1)	10.5 (9.3; 11.6)	3.6 (3.2; 4.1)
Goiânia	58.2 (56.6; 59.9)	15.2 (13.7; 16.6)	1.1 (0.9; 1.3)
Brasília	72.7 (70.3; 75.0)	4.8 (3.8; 5.7)	0.6 (0.5; 0.7)

* TB = tuberculosis;

† 95%CI = 95% Confidence Interval;

‡ rates of cure and abandonment calculated in percentage up to 2014;

§ Mortality coefficients per 100,000 inhabitants up to 2015

Source: Sinan-TB, SIM, IBGE, 2001-2015

For the analysis of the trends of the three outcome situations analyzed,the AAPC at the end of the periods was considered. After this analysis, it was possible to identify an expressive heterogeneity throughout the national territory, even in states belonging to the same region. Nationwide, 22% (6) capitals showed a significant increase in the annual cure rate, and 15% (4) presented a decrease. The remainder (63%; 17) did not present statistical significance, being interpreted as a trend stationary.

The two capitals that deserve to be highlighted concerning the cure of the disease are Campo Grande, with a decrease of 3% per year in the cure rate (95%CI: −5.0 - −0.9), and Rio de Janeiro, with an average increase of 3.5% (95%CI: 1.9 - 4.7). In the North region, only Rio Branco showed a significant increase in the annual cure rate rate (AAPC: 1.6; CI: 0.7 - 2.5). On the other hand, Porto Velho showed a statistically significant reduction (AAPC: −1.4; CI: −2.4 - −0.3). In the Northeast region, Salvador was the only capital with an increase in the cure rate (AAPC: 1.1; CI: 0.1 - 2.1), and Natal with a decrease in it (AAPC: −2.3; CI: −3.8 - −0.7). It was not possible to find significant changes in any capital of the South region (Table 2).

**Table 2:** Annual Percentage Change of the cure outcome of TB* treatment in Brazilian capitals from 2001 to 2014

Capitals	APC1^†^ (95%CI)^||^	IP^‡^	APC2^†^ (95%CI)^||^	IP^‡^	APC3^†^ (95%CI)^||^	AAPC^§^ (95%CI)^||^
North Region						
Porto Velho	-1.4 (-2.4; -0.3)					-1.4 (-2.4; -0.3)^¶^
Rio Branco	1.6 (0.7; 2.5)					1.6 (0.7; 2.5)^¶^
Manaus	-3.7 (-5.6; -1.8)	2007	6.7 (-6.0; 21.1)	2010	-2.5 (5.8; 1.0)	-1.0 (-3.5; 1.6)
Boa Vista	-0.1 (-1.1; 1.0)					-0.1 (-1.1; 1.0)
Belém	0.3 (-0.5; 1.2)					0.3 (-0.5; 1.2)
Macapá	-0.3 (-3.5; 3.0)	2006	4.6 (1.8; 7.8)	2012	-2.9 (-13.3; 8.7)	1.5 (-0.5; 3.6)
Palmas	6.2 (0.7; 12.1)	2008	-3.7 (-8.8; 1.6)			1.5 (-1.8; 4.9)
Northeast Region						
São Luís	-0.3 (-0.9; 0.4)					-0.3 (-0.9; 0.4)
Teresina	-2.1 (-7.3; 3.3)	2008	17.7 (-1.2; 40.2)	2012	-6.1 (-29.7; 25.4)	2.9 (-3.1; 9.4)
Fortaleza	-0.0 (-2.4; 2.5)					-0.0 (-2.4; 2.5)
Natal	-13.2 (-22.2; -3.2)	2003	-0.1 (-1.1; 0.8)			-2.3 (-3.8; -0.7)^¶|^
João Pessoa	-0.5 (-1.7; 0.7)					-0.5 (-1.7; 0.7)
Recife	2.0 (0.6; 3.4)	2006	-4.6 (-10.3; 1.4)	2009	1.3 (-0.1; 2.7)	0.2 (-1.1; 1.5)
Maceió	-1.0 (-2.0; 0.1)					-1.0 (-2.0; 0.1)
Aracaju	-0.7 (-2.1; 0.6)					-0.7 (-2.1; 0.6)
Salvador	1.1 (0.1; 2.1)					1.1 (0.1; 2.1)^¶^
Southeast Region						
Belo Horizonte	0.0 (-0.6; 0.7)					0.0 (-0.6; 0.7)
Vitória	2.0 (0.8; 3.2)					2.0 (0.8; 3.2)^¶^
Rio de Janeiro	3.5 (1.9; 4.7)					3.5 (1.9; 4.7)^¶^
São Paulo	5.7 (-0.7; 12.5)	2004	0.1 (-0.7; 1.0)			1.4 (0.0; 2.8)^¶^
South Region						
Curitiba	-1.8 (-4.9; 1.4)	2007	7.9 (-2.0; 18.9)	2011	-3.5 (-12.0; 5.9)	0.7 (-2.4; 3.9)
Florianópolis	-0.0 (-2.5; 2.5)					-0.0 (-2.5; 2.5)
Porto Alegre	4.1 (-0.4; 8.8)	2004	-1.8 (-2.4; -1.1)			-0.4 (-1.4; 0.6)
Midwest Region						
Campo Grande	2.1 (-2.3; 6.6)	2006	-6.0 (-8.6; -3.2)			-3.0 (-5.0; -0.9)^¶^
Cuiabá	-2.0 (-2.4; -1.7)					-2.0 (-2.4; -1.7)^¶^
Goiânia	0.1 (-0.6; 0.8)					0.1 (-0.6; 0.8)
Brasília	0.8 (0.1; 1.5)					0.8 (0.1; 1.5)^¶^

* TB = tuberculosis;

† APC = Annual Percentage Change;

‡ IP = Inflection Point (year in which the line segment changes);

§ AAPC = Average Annual Percentage Change;

|| 95%CI = 95% Confidence Interval;

¶ p<0.05

Source: Sinan-TB, 2001-2014.

The next outcome, treatment abandonment, presented expressive increasing rates. Of the 26 cities studied (Palmas could not be analyzed due to lack of data in some years during the series), half (13) significantly increased the abandonment rate and only 4% (1) decreased this rate over the years. Of these capitals, 46% (12) showed a trend stationary at the end of the series.

The most expressive results are those of Rio Branco, which showed an average decrease of 10.9% per year in the rate of treatment abandonment (95%CI: −15.8 - −5.7), and Fortaleza, which presented an average increase of 12.8% per year (95%CI: 7.6 - 18.3). In addition, in the North region, only Rio Branco reduced its abandonment rate after the 14 years (AAPC: −10.9; 95%CI: −15.8 - −5.7). In the Northeast, no capital showed a significant decrease in the rate at the end of the series. The same situation occurs in the other regions (Southeast, South, and Midwest) (Table 3).

**Table 3:** Annual Percentage Change of the outcome abandonment of TB* treatment in Brazilian capitals from 2001 to 2014

Capitals	APC1^†^ (95%CI)^||^	IP^‡^	APC2^†^ (95%CI)^||^	IP^‡^	APC3^†^ (95%CI)^||^	AAPC^§^ (95%CI)^||^
North Region						
Porto Velho	5.3 (3.2; 7.5)					5.3 (3.2; 7.5)^¶^
Rio Branco	-10.9 (-15.8; -5.7)					-10.9 (-15.8; -5.7)^¶^
Manaus	2.5 (1.2; 3.9)					2.5 (1.2; 3.9)^¶^
Boa Vista	6.4 (2.0; 11.0)					6.4 (2.0; 11.0)^¶^
Belém	4.1 (0.5; 7.8)	2008	-12.4 (-32.6; 13.8)	2011	4.5 (-9.2; 20.3)	0.1 (-5.4; 6.0)
Macapá	-9.1 (-17.1; -0.4)	2003	13.3 (-4.4; 34.2)	2012	-27.9 (-62.5; 38.7)	-4.5 (-13.8; 5.7)
Palmas**						
Northeast Region						
São Luís	-16.4 (-25.0; -6.8)	2006	27.2 (-28.0; 124.7)	2009	-3.3 (-12.6; 6.9)	-2.6 (-13.2; 9.2)
Teresina	-24.8 (-56.4; 29.4)	2003	6.8 (2.5; 11.2)			1.2 (-6.5; 9.4)
Fortaleza	51.0 (6.5; 114.3)	2003	7.0 (5.6; 8.4)			12.8 (7.6; 18.3)^¶^
Natal	-2.8 (-6.8; 1.3)					-2.8 (-6.8; 1.3)
João Pessoa	4.5 (1.4; 7.6)					4.5 (1.4; 7.6)^¶^
Recife	-3.4 (-6.8; 0.1)	2007	15.5 (-4.8; 40.1)	2010	-5.4 (-10.8; 0.4)	0.0 (-4.0; 4.2)
Maceió	1.2 (-0.3; 2.6)					1.2 (-0.3; 2.6)
Aracaju	4.2 (1.5; 7.0)					4.2 (1.5; 7.0)^¶^
Salvador	2.9 (2.1; 3.8)					2.9 (2.1; 3.8)^¶^
Southeast Region						
Belo Horizonte	2.2 (0.1; 4.3)					2.2 (0.1; 4.3)^¶^
Vitória	3.7 (0.8; 6.8)					3.7 (0.8; 6.8)^¶^
Rio de Janeiro	10.3 (5.3; 15.5)	2009	-5.7 (-13.5; 2.8)			3.8 (-0.0; 7.8)
São Paulo	-0.1 (-0.9; 0.7)					-0.1 (-0.9; 0.7)
South Region						
Curitiba	-2.5 (-5.0; 0.0)					-2.5 (-5.0; 0.0)
Florianópolis	1.4 (-1.0; 3.9)					1.4 (-1.0; 3.9)
Porto Alegre	0.4 (-6.0; 7.2)	2006	12.7 (7.2; 18.5)	2012	-0.9 (-16.6; 17.6)	5.7 (2.1; 9.4)^¶^
Midwest Region						
Campo Grande	6.7 (3.0; 10.6)					6.7 (3.0; 10.6)^¶^
Cuiabá	2.3 (0.2; 4.4)					2.3 (0.2; 4.4)^¶^
Goiânia	3.2 (1.7; 4.7)					3.2 (1.7; 4.7)^¶^
Brasília	-8.9 (-14.4; -2.9)	2010	23.8 (-1.1; 54.9)			0.1 (-6.7; 7.5)

* TB = Tuberculosis;

† APC = Annual Percentage Change;

‡ IP = Inflection Point (year in which the line segment changes);

§ AAPC = Average Annual Percentage Change;

|| 95%CI = 95% Confidence Interval;

¶ p<0.05;

** Palmas data could not be analyzed due to lack of information in some years

Source: Sinan-TB, 2001-2014

The last outcome evaluated, mortality by TB, has been decreasing over time throughout Brazil. For this analysis, 25 capitals were studied (Boa Vista and Palmas were not analyzed due to lack of data in the time series), and 44% (n=11) of them significantly reduced their AAPC, thus, the mortality by TB. The other capitals (56%; 14) presented a trend stationary, and no significant increase in TB mortality was detected over the years.

Thus, the highest decrease in mortality was found in Porto Velho (AAPC: 7.8; 95%CI: −11.0 - −5.0) and the lowest one in Porto Alegre (AAPC: 2.5; 95%CI: −4.5 - −0.6). In addition, in the Northeast region, Teresina presented the highest coefficient reduction (AAPC: −4.2; 95%CI: −7.4 - −0.8). In the Southeast region, São Paulo stood out in the reduction of mortality (AAPC: −3.5; 95%CI: −4.9 - −2.1). Finally, Brasília was the capital of the Midwest region with the highest decrease in the average mortality rate (AAPC: −4.6; 95%CI: −7.9 - −1.1) (Table 4).

**Table 4:** Annual Percentage Variation of the mortality coefficient of TB* in Brazilian capitals from 2001 to 2015

Capitals	APC1^†^ (95%CI)^||^	IP^‡^	APC2^†^ (95%CI)^||^	IP^‡^	APC3^†^ (95%CI)^||^	AAPC^§^ (95%CI)^||^
North Region						
Porto Velho	-7.8 (-11.0; -5.0)					-7.8 (-11.0; -5.0)^¶^
Rio Branco	-5.7 (-9.1; -2.2)					-5.7 (-9.1; -2.2)^¶^
Manaus	-0.8 (-2.8; 1.1)					-0.8 (-2.8; 1.1)
Boa Vista**						
Belém	0.6 (-2.0; 3.2)					0.6 (-2.0; 3.2)
Macapá	1.0 (-3.3; 5.6)					1.0 (-3.3; 5.6)
Palmas**						
Northeast Region						
São Luís	1.9 (-1.0; 4.9)	2011	-10.8 (-21.2; 1.0)			-1.9 (-5.4; 1.7)
Teresina	-4.2 (-7.4; -0.8)					-4.2 (-7.4; -0.8)^¶^
Fortaleza	-21.8 (-39.1; 0.4)	2003	17.5 (3.7; 33.1)	2007	-5.7 (-8.2; -5.4)	-2.3 (-6.4; 2.1)
Natal	0.2 (-2.8; 3.4)					0.2 (-2.8; 3.4)
João Pessoa	1.3 (-1.6; 4.3)					1.3 (-1.6; 4.3)
Recife	-3.0 (-4.3; -1.7)					-3.0 (-4.3; -1.7)^¶^
Maceió	0.1 (-2.0; 2.3)					0.1 (-2.0; 2.3)
Aracaju	2.6 (-0.9; 6.1)					2.6 (-0.9; 6.1)
Salvador	-3.5 (-5.3; -1.6)					-3.5 (-5.3; -1.6)^¶^
Southeast Region						
Belo Horizonte	-2.8 (-5.4; -0.2)					-2.8 (-5.4; -0.2)^¶^
Vitória	-2.2 (-7.4; 3.3)					-2.2 (-7.4; 3.3)
Rio de Janeiro	-6.2 (-12.0; -0.0)	2005	1.6 (0.0; 3.3)			-0.7 (-2.5; 1.2)
São Paulo	-8.5 (-12.7; -4.2)	2005	-1.4 (-2.7; -0.2)			-3.5 (-4.9; -2.1)^¶^
South Region						
Curitiba	-6.2 (-9.0; -3.2)					-6.2 (-9.0; -3.2)^¶^
Florianópolis	0.5 (-5.2; 6.5)					0.5 (-5.2; 6.5)
Porto Alegre	-2.5 (-4.5; -0.6)					-2.5 (-4.5; -0.6)^¶^
Midwest Region						
Campo Grande	-0.5 (-3.8; 2.9)					-0.5 (-3.8; 2.9)
Cuiabá	-0.1 (-3.0; 2.8)					-0.1 (-3.0; 2.8)
Goiânia	-1.0 (-5.7; 3.9)					-1.0 (-5.7; 3.9)
Brasília	-4.6 (-7.9; -1.1)					-4.6 (-7.9; -1.1)^¶^

* TB = Tuberculosis (coefficient calculated for 100,000 inhabitants);

† APC = Annual Percentage Change;

‡ IP = Inflection Point (year in which the line segment changes);

§ AAPC = Average Annual Percentage Change;

|| 95%CI = 95% Confidence Interval;

¶ p<0.05;

** Boa Vista and Palmas data could not be analyzed due to lack of information in some years

Source: Sinan-TB, SIM, IBGE, 2001-2015.

## Discussion

The main results lead us to the finding that the cure is stationary, while treatment abandonment showed a growth trend and mortality, a decrease trend. The limitation of this study occurs mainly because of its secondary-based situation, which may restrict the external validity of the data, and by the change in the notification and follow-up form of the disease in 2014.

Concerning the interpretation of results related to cure, the national database study^(^ 18 ^)^ suggests that TB cure rates have decreased, even in the presence of new interventions, such as the inclusion of ethambutol (E) in the former rifampicin, isoniazid, pyrazinamide (RHZ) treatment. New cases present better cure rates compared to retreatment or return after abandonment^(^ 19 ^)^. The literature points out that knowledge, ease of access to the health service, adequate infrastructure, health education, availability of drugs, and patient-focused care, with respect to their sociocultural characteristics, promote the cure^(^ 20 ^-^ 21 ^)^.

Regarding the interpretation of results related to treatment abandonment, this study indicates that the reduction of cure is intimately associated with increased treatment abandonment^(^ 18 ^)^. The following factors are associated with treatment abandonment: male sex; retreatment; use of illegal drugs; malnutrition; few years of schooling; lack of knowledge about the disease; access to health service^(^ 22 ^-^ 24 ^)^; and coinfection by the Human Immunodeficiency Virus (HIV). This decreases by 58% the chances of cure of a TB patient, increases by 50% the chances of abandonment and by 94% the chances of death by TB^(^ 12 ^)^, increasing the notifications in the information systems^(^ 25 ^)^. It was shown that, as much as the abandonment rate is within the one recommended by the WHO, the cases of coinfection must be monitored to avoid new negative outcomes^(^ 26 ^)^. Moreover, the expansion of drug treatment coverage to people living with HIV, along with preventive measures, has the potential to reduce the TB burden^(^ 27 ^)^.

This study has shown a significant increase in the abandonment of TB treatment in most national capitals. As pointed out in the literature, a factor that may contribute to this outcome is the lack of qualified and structured care, because the search for more distant units, but that are references in TB care, is still common among patients, who travel longer itineraries to develop their treatment^(^ 28 ^)^. It is believed that the decentralization of health services specialized in caring for people with TB promotes quality care and the supervised treatment of patients, which could decrease abandonment.

In addition, cases classified as retreatment have twice as much chance of new abandonment, as well as those with negative bacilloscopy and unknown HIV status^(^ 29 ^)^. The literature indicates that the reduction of extreme poverty would decrease the incidence of TB by 33.4% up to 2035, and that the increase in social protection would reduce it by 76.1% up to the same year^(^ 30 ^)^.

In this context, it is worth noting the importance of the Directly Observed Therapy (DOT) in the TB care. The DOT, component of the End TB Strategy, is another approach in the treatment of people with TB, especially those with more chances of abandoning treatment, as it accompanies the taking of medications (daily or alternating), a resource used to expand the adherence to drug treatment^(^ 31 ^)^. According to Datasus, 20 of the 27 capitals studied had a coverage of DOT lower than 50%, data that may be a possible explanation for the growth in the numbers of abandonment in Brazil. Even so, although the DOT approximates patient and health sector, there is still difficulty in shaping its real impact on the success of TB treatment^(^ 32 ^)^.

The failure of these programs can lead the patient to develop drug-resistant TB (DR-TB). DR-TB is one of the current problems faced by countries in the management of the disease. For people with drug-resistant TB, the literature is inconclusive about treatment outcomes in those who follow DOT, compared to those in self-administered treatment, since clinical and non-clinical studies have found disparate results. However, a systematic review and meta-analysis of observational studies showed that 63.5% of them conclude treatment with a 55.6% cure rate, 14.2% abandonment rate, and 12.6% death rate. Therefore, there is a need for strategies to reduce treatment abandonment and drug resistance^(^ 33 ^)^.

Regarding mortality, it had decreased across the world in people without HIV infection^(^ 34 ^)^, and has the following associated factors: male sex; lower economic power; lower Human Development Index (HDI); higher immigration rate; black ethnicity and higher coinfection coefficients for HIV^(^ 10 ^,^ 13 ^,^ 35 ^-^ 36 ^)^; vulnerable (immunosuppressed) populations, people with circulatory problems and neoplasia; and people with delayed diagnosis and inefficient follow-up^(^ 37 ^)^. The decrease in mortality observed in this study may derive from public policies that prioritize the monitoring of the patient, free treatment in the appropriate time, and social protection measures with money transfer^(^ 38 ^)^. The investigation of all national capitals has shown data for analysis, which can subsidize new policies to increase cure and reduce unfavorable outcomes.

## Conclusion

When analyzing the temporal pattern of the outcomes cure, abandonment, and mortality by TB in Brazilian capitals, this study showed a trend stationary for cure, growth for abandonment, and decrease for mortality. Brazilian capitals were not uniform regarding the outcome situation of TB treatment. The results of this research are important for disease management programs at the local and national levels. It is clear that Brazilian capitals need more efficient interventions to reduce negative outcomes and improve the cure rate of the disease, as the End TB Strategy points out.
